# Tailored Web-Based Information for Younger and Older Patients with Cancer: Randomized Controlled Trial of a Preparatory Educational Intervention on Patient Outcomes

**DOI:** 10.2196/14407

**Published:** 2019-10-01

**Authors:** Minh Hao Nguyen, Ellen M A Smets, Nadine Bol, Eugène F Loos, Hanneke W M van Laarhoven, Debby Geijsen, Mark I van Berge Henegouwen, Kristien M A J Tytgat, Julia C M van Weert

**Affiliations:** 1 Amsterdam School of Communication Research University of Amsterdam Amsterdam Netherlands; 2 Department of Communication and Media Research (IKMZ) University of Zurich Zurich Switzerland; 3 Department of Medical Psychology Amsterdam University Medical Centers University of Amsterdam Amsterdam Netherlands; 4 Department of Communication and Cognition Tilburg University Tilburg Netherlands; 5 Department of Medical Oncology, Cancer Center Amsterdam Amsterdam University Medical Centers University of Amsterdam Amsterdam Netherlands; 6 Department of Radiation Oncology Amsterdam University Medical Centers University of Amsterdam Amsterdam Netherlands; 7 Department of Surgery, Cancer Center Amsterdam Amsterdam University Medical Centers University of Amsterdam Amsterdam Netherlands; 8 Department of Gastroenterology Amsterdam University Medical Centers University of Amsterdam Amsterdam Netherlands

**Keywords:** Web-based tailoring, internet, audiovisual media, patient education, cancer, aging, memory, anxiety, patient reported outcomes, patient participation, consultation, health communication, randomized controlled trial

## Abstract

**Background:**

Many patients with cancer, including older patients (aged ≥65 years), consult the Web to prepare for their doctor’s visit. In particular, older patients have varying needs regarding the mode in which information is presented (eg, via textual, visual, or audiovisual modes) owing to age-related sensory (eg, impaired vision and hearing) and cognitive decline (eg, reduced processing speed). Therefore, Web-based information targeted at older patient populations is likely to be used and processed more effectively, and evaluated more positively, when tailored to age-related capabilities and preferences. This, in turn, may benefit patient outcomes.

**Objective:**

This randomized controlled trial tested the effects of a Web-based tailored educational intervention among newly diagnosed younger (<65 years) and older (≥65 years) patients with cancer. We compared the intervention group who viewed a mode-tailored website (ie, enabling patients to tailor information using textual, visual, and audiovisual modes) with 3 control groups view a nontailored website (ie, text only, text with images, and text with videos). We examined website experience outcomes (ie, website satisfaction, website involvement, knowledge, anxiety, and communication self-efficacy) and consultation experience outcomes (ie, question asking during consultation, anxiety, and information recall).

**Methods:**

Patients from a multidisciplinary outpatient clinic (N=232) viewed a mode-tailored or nontailored website as preparation before their hospital consultations to discuss diagnosis and treatment. Data were collected before (T1), during (T2), and after (T3) visitation. Website experience outcomes were assessed with questionnaires (T1). Patients’ question asking was coded from videotaped consultations, and anxiety was assessed through a questionnaire (T2). Telephone interviews were conducted to assess knowledge acquired from the website before (T1) and after consultation (T3), and information recall from the consultation (T3).

**Results:**

The preparatory website was well used across all conditions (mean 34 min). Younger patients viewing the mode-tailored website were more satisfied before consultation (*P*=.02) and reported lower anxiety after consultation (*P*=.046; vs text only). This pattern was not found in older patients. Mode tailoring yielded no other significant differences in patient outcomes. Regression analyses showed that website involvement (beta=.15; *P*=.03) and, to a lesser extent, website satisfaction (beta=.15; *P*=.05) positively associated with knowledge before consultation (T1). In turn, higher knowledge before consultation (beta=.39; *P*<.001), together with time on the website (beta=.21; *P*=.002; T1), predicted information recall from consultations (T3). Patients with higher knowledge before consultation (T1) also reported higher knowledge from the website afterward (T3; beta=.22; *P*=.003).

**Conclusions:**

Offering preparatory online information before consultations benefits information processing and patient outcomes of both younger and older newly diagnosed patients with cancer. Younger patients benefit even more when information is offered in a mode-tailored manner. We discuss the theoretical, methodological, and practical implications for patient-provider communication research in an electronic health era.

**Clinical Trial:**

Netherlands Trial Register NTR5904; https://www.trialregister.nl/trial/5750

## Introduction

### Background

Cancer often occurs in people of older age (≥65 years), and this number is expected to grow globally [1]. Older patients with cancer constitute the majority of the cancer patient population and are also most at risk for poor communication with health care providers owing to age-related declines, such as in cognitive (eg, working memory) and physical functioning (eg, vision loss, hearing loss, and comorbidity) [2]. In general, older patients are less likely to express their information needs or preferences and participate less actively during consultations [2,3]. Moreover, they generally experience lower self-efficacy in obtaining relevant information from their provider [4] and have more difficulty remembering information from consultations than younger patients [5,6]. Therefore, particularly, older patients could benefit from support by communicating with providers. This study aimed to investigate whether tailored online health information can provide such support to older and younger patients by examining the effects on patient-reported and observed outcomes, including website satisfaction, communication self-efficacy, anxiety, question asking during consultation, and information recall.

The information society of today is characterized by the availability of and relatively easy access to cancer information on the internet. For many older adults, besides their health care provider, the internet is one of the first preferred health information sources [7]. Online health information (eg, a hospital website) is often used to prepare for a doctor’s visit [7] and may lead to better informed, more confident, and less anxious patients [8,9]. Moreover, the use of preparation tools can support patients to actively participate in consultations (eg, by asking questions) and process and recall information from their health care provider [10,11].

Unfortunately, many older patients experience difficulties in using online health information [12]. Although this problem could resolve itself as generations pass by and the digital divide closes, age-related sensory (eg, impaired vision and/or hearing) and cognitive decline (eg, reduced processing speed) remain a prominent reason preventing older adults from using the internet effectively [12,13]. Such age-related declines also explain why older adults have varying needs regarding *how* information should be presented, making it more challenging to develop user-friendly websites for this group [14,15]. Online health information distinguishes itself from traditional formats of health information (eg, print materials) because of its possibility to integrate different modalities (ie, modes), such as textual, visual, and/or audiovisual information. What is particularly relevant for older patients is that these information modes can be tailored to match individual preferences and abilities (eg, age-related factors) and thus facilitate information processing [16]. *Mode tailoring* refers to the possibility of individuals to adapt the modality of online information presentation to their preferences, using textual, visual, and audiovisual information [17]. Recent experimental research showed positive effects on the evaluation, processing, and recall of cancer-related information when people were able to self-tailor the mode of presentation on a health website, especially among older adults [17,18]. Hence, mode tailoring is a particularly promising strategy to optimize online health information for the older population.

This study extends this experimental mode tailoring research to a clinical population of newly diagnosed patients with cancer who viewed a previsit website to prepare for their hospital consultations to discuss diagnosis and treatment planning. In a randomized controlled trial (RCT), we investigated both pre- and postvisit effects of exposure to a previsit website that can be tailored to patients’ information mode preferences (by self-selecting text, images, and/or videos) compared with exposure to standardized, nontailored websites (with either text only, text with images, or text with videos). First, we examined the effects of mode tailoring on website experience outcomes before the consultation (T1), including patients’ website involvement, satisfaction with the website, anxiety, self-efficacy in communicating with the provider, and knowledge. We also investigated whether mode tailoring effects extend to the consultation and beyond. Consultation experience outcomes include patients’ question-asking behavior during consultation and anxiety (T2). Additionally, we considered knowledge gained from the website and information recall after the consultation (T3). Second, for all outcomes, we investigated how these effects differ between younger and older patients. Third, across all patients, we investigated how website experiences predict knowledge before the consultation (T1) and how website experiences and consultation experiences predict knowledge and information recall after the consultation (T3). By looking into the interplay between online health information provision and offline patient-provider communication in the cancer context, this study has provided insights for both practice and theory regarding patient-provider communication in an electronic health (eHealth) era.

### Mode Tailoring: Catering to Older Patients’ Motivation and Ability

The elaboration likelihood model (ELM) and the limited capacity model of motivated mediated message processing (LC4MP) state that information processing is highly dependent on an individual’s motivation (eg, attention) and ability (eg, cognitive resources) to process information [19,20]. Older adults often see themselves as less able and are less motivated to use online health information [21]. Moreover, many older adults who go online for health information are left unsatisfied [22]. A partial explanation is that many available health websites insufficiently consider age-related factors in their design [12,23]. Providing different information modes (eg, via text, visuals, and videos) in a tailored manner can increase both the *motivation* and *ability* to use and process online health information and may, therefore, be especially relevant for older users. For instance, when the mode of presentation matches with an individual’s *preference* for how to consume online health information, this is likely to increase their *motivation* to attend to the information. Additionally, tailoring the mode of information presentation caters to differences in individual processing styles and abilities—including age-related declines in vision, hearing, and cognition—which *enables* individuals to process the information better. Thus, when online health information is tailored to individual mode preferences, these preconditions (ie, motivation and ability) for successful processing are considered more optimal. Consequently, mode-tailored information has a greater likelihood to reach and affect patients than nontailored information, especially older patients. In the following sections, we have discussed the expected benefits of mode-tailored online health information for patient-reported outcomes surrounding a hospital visit in younger and older patients.

### Effects on Website Experience Outcomes Before Consultation: Involvement, Satisfaction, Anxiety, Communication Self-Efficacy, and Knowledge

A (potential) diagnosis of cancer typically involves high levels of anxiety [24], which can hinder patients’ ability to process and remember information provided by their provider [18]. Although it can be overwhelming to receive information related to the disease [25], patients have a high need for information during this uncertain phase [26]. Providing patients with mode-tailored information might enable them to absorb the information in a dosed manner (eg, by reading the text first and saving a video for later) [27]. As mode-tailored information is more accessible to patients, it is expected to be evaluated and processed better too [17,18]. Furthermore, viewing tailored online information before a hospital visit is likely to decrease patients’ anxiety, as they are better informed and prepared for what can be expected [28]. Similarly, the use of tailored preparatory information might increase self-efficacy in communicating with the provider [29,30]. Given that mode tailoring is anticipated to cater to age-related declines, we expect that older patients will benefit more from a mode-tailored website than younger patients.

- H1: Exposure to a mode-tailored preparatory website (vs nontailored websites) will affect patients’ website experience outcomes before a consultation (T1), including enhanced website involvement (H1a); enhanced website satisfaction (H1b); decreased anxiety (H1c); enhanced communication self-efficacy (H1d); and improved knowledge (H1e).
- H2: These effects will be stronger for older patients (≥65 years) than for younger patients (<65 years) with regard to website involvement (H2a), website satisfaction (H2b), anxiety (H2c), communication self-efficacy (H2d), and knowledge (H2e).

### Effects on Consultation Experience Outcomes: Question Asking and Anxiety

The abovementioned effects of mode-tailored online information might also extend to the consultation and beyond (eg, patients’ question asking and anxiety). Combining tailored preparatory information and interpersonal patient-provider communication can reinforce each other’s effectiveness [31]. For instance, viewing preparatory information may make patients aware of topics of information they would like to know more about or validate with their health care provider, causing them to be more actively involved during consultations by asking questions [30,32]. Alternatively, patients viewing preparatory information before consultation may feel better informed and prepared for their visit, resulting in fewer questions asked during consultation [29]. Regardless, providing preparatory information in a tailored manner could strengthen effects in both directions (ie, more or less questions). As it is unclear how viewing mode-tailored online information before consultation would affect patients’ question asking and how this differs by age, the following research questions were formulated:

RQ1: Does exposure to a mode-tailored preparatory website (vs nontailored websites) lead to more or less questions asked by patients during consultation (T2)?

RQ2: To what extent does the relation between exposure to a mode-tailored preparatory website and patients’ question asking during consultation differ between younger (<65 years) and older patients (≥65 years)?

Preparatory information can play a key role in limiting anxiety during cancer consultations, perhaps even more so for patients who tend to avoid information [24]. Bronner et al found that patients with cancer characterized by a monitoring coping style, that is, *information seekers*, became less anxious from pre- to postconsultation after receiving their diagnosis and treatment plan [24]. The opposite relation was found for patients identified more as *information avoiders*; this group became more anxious from pre- to postconsultation, especially when receiving bad news [24]. A possible explanation is that information seekers had already searched for information before their consultation and were prepared for the worst scenario. Thus, when hearing their diagnosis and treatment advice, they felt relieved when hearing relatively *good* news or they were more prepared for bad news. This is in contrast with the *less prepared* information avoiders who became more distressed after their consultation when receiving bad news. Therefore, we expect that the use of preparatory information before hospital visits, especially when tailored, decreases anxiety immediately after consultation.

- H3: Exposure to a mode-tailored preparatory website (vs nontailored websites) will decrease anxiety immediately after consultation (T2).

Additionally, we expect the effect of mode-tailored preparatory information on anxiety to be especially visible in older patients. The socioemotional selectivity theory posits that as people age, goals associated with emotional meaning and well-being become more salient, whereas knowledge-related goals to prepare for future events become less important [33]. Consequently, older adults generally process information in such a way that it helps them regulate their emotions (eg, putting them at ease). Older patients may perceive more emotional gratification from information presented in visual and audiovisual modes because these modes often include more vivid and obvious personal elements (eg, a patient video) that appeal more to their emotion-oriented preferences and needs. For instance, previous research has shown that older adults often prefer visual and audiovisual information [15,34], and such information modes have been found to increase feelings of emotional support from online cancer-related information compared with text in older people [35,36]. However, studies have also shown high variability in older adults’ information mode preferences [12,37]. As such, providing older patients with the option to select their preferred information modes, including visual and audiovisual elements, is more likely to fulfil their emotional and informational needs, thereby limiting their anxiety.

- H4: The effect of mode tailoring on anxiety immediately after consultation will be stronger for older patients (≥65 years) than for younger patients (<65 years).

### Effects on Knowledge and Recall of Information After the Consultation

Consulting online information before consultations might also improve knowledge from online information and information recall after the consultation. For instance, when patients are already informed about several topics before consultations, this could prime patients’ attention to these and related topics when being discussed by the provider during consultations (ie, a repetition effect) [38]. Additionally, being informed and knowing what to expect beforehand could leave patients with more cognitive capacity to attend to new information that they receive during consultations. In other words, providing information in a dosed manner over multiple occasions allows patients to process important information at a slower pace which may benefit information recall [39]. As older patients have more difficulty in remembering medical information, it is expected that they will benefit relatively more from mode-tailored preparatory information than younger patients.

- H5: Exposure to a mode-tailored preparatory website (vs nontailored websites) will improve knowledge from the website and information recall from the consultation (T3).
- H6: The effect on knowledge from the website and information recall from the consultation will be stronger for older patients (≥65 years) than for younger patients (<65 years).

### Motivation- and Ability-Related Factors Explaining Patients’ Information Processing

Besides the main effects of mode tailoring on patient-reported outcomes before, during, and after the consultations, different *website experience outcomes* (eg, website involvement) may independently explain knowledge before the consultation (T1) and, together with *consultation experience outcomes* (T2; eg, question asking and anxiety), predict knowledge from the website and information recall from the consultation (T3). The different processes explaining knowledge acquisition and information recall can be related to a patient’s *motivation* (eg, website involvement) or *ability* (eg, communication self-efficacy) to process information. Although ELM and LC4MP are useful frameworks in understanding how mode tailoring can enhance *motivation* and *ability* to process information, how these processes translate to specific variables explaining knowledge and recall of information in the cancer context has only been briefly explored [5,6,40]. For instance, Bol et al used ELM and LC4MP to identify motivation- and ability-related factors in the literature deemed relevant for processing of online cancer information [40]. They identified website involvement and website satisfaction as *website experience outcomes* positively associated with information recall, whereas perceived cognitive load was negatively related to information recall. However, Bol et al did not examine which *consultation experience outcomes* contribute to effective information processing in patient-provider encounters [40]. Thus, in addition to addressing the value of mode tailoring, this study sought to gain insight into which *website experience outcomes* and *consultation experience outcomes* explain the benefits of preparatory online information on knowledge acquisition from websites and information recall from consultations in patients with cancer, as well as how these concepts relate to each other over time. By doing so, we inform future research relying on theories such as the ELM and LC4MP to understand which specific *motivation*- and *ability*-related factors play a role in how online and offline cancer information is being processed. We explored the following research questions:

RQ3: Which website experience outcomes (eg, website use, website involvement, and anxiety) predict knowledge from the website before a consultation (T1)?

RQ4: Which website experience outcomes (T1) and which consultation experience outcomes (ie, question asking and anxiety; T2) predict knowledge from the website and information recall after the consultation (T3)?

## Methods

### Design

An RCT was conducted to compare the effectiveness of a mode-tailored website (with options to choose text, visuals, and/or videos) with 3 standardized, nontailored versions with text only, text with visuals, and text with videos. Patients were stratified into a younger group (<65 years) and older group (≥65 years) and randomly assigned to view one of the 4 website versions. An age cut-off of 65 years was selected, as cancer more frequently occurs in adults above this age [1] and as similar studies on older patients and online health information also used this cut-off age [12,35,36]. An *a priori* power analysis based on an analysis of variance (ANOVA) with 8 groups (condition × age group) revealed that a sample size of 237 was needed to detect a medium-sized effect (Cohen *f*=0.25) with an observed power of 0.80 and an alpha level of .05. The study was approved by the medical ethical review board of the Amsterdam University Medical Center (reference number: W13_053 #13.17.0069) and the ethics committee of the Amsterdam School of Communication Research (reference number: 2014-CW-110).

### Participants

**Figure 1:**
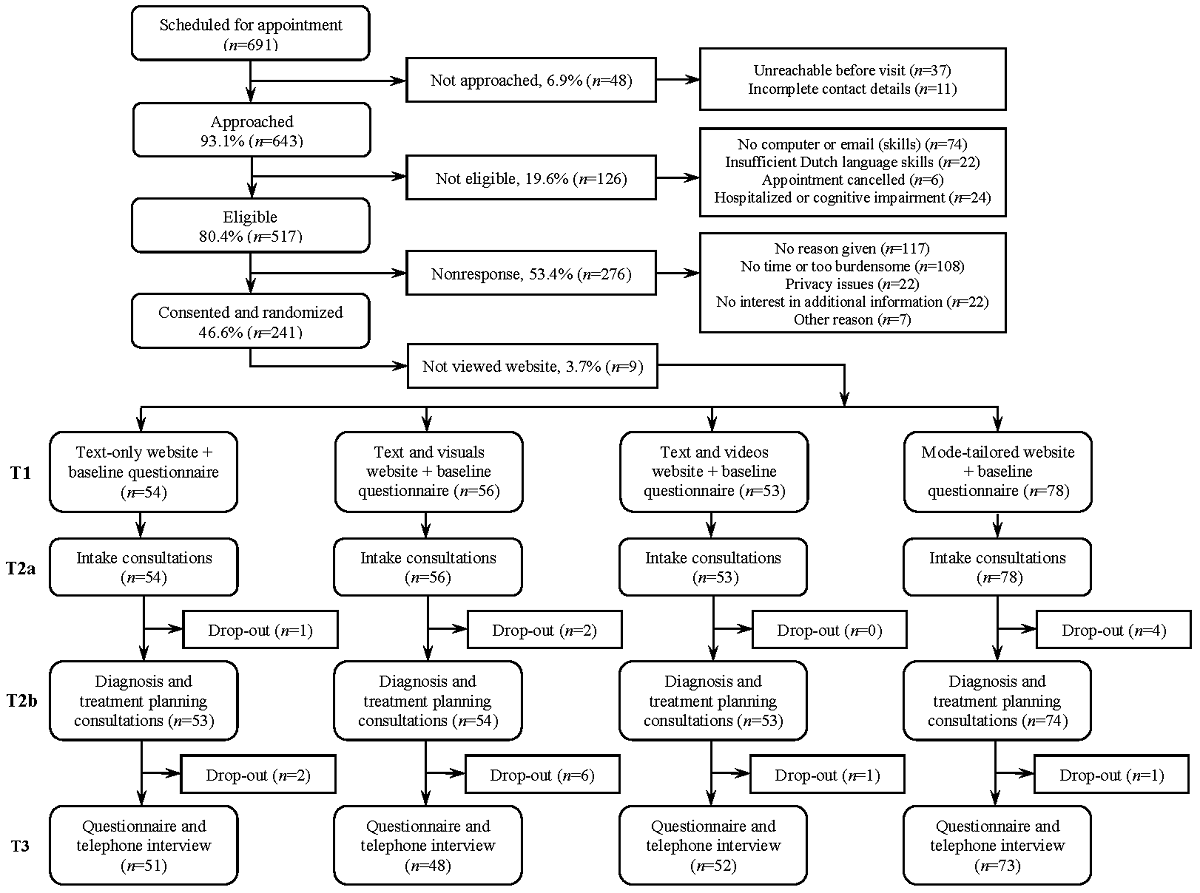
Flowchart of participant recruitment and drop-out.

Participants were patients who were suspected of having colorectal, stomach, or esophageal malignancies or had received a preliminary cancer diagnosis (but were awaiting information on the tumor stage based on additional imaging or came for a second opinion) who were referred to the Gastro-Intestinal Oncological Centre Amsterdam (GIOCA). Patients were recruited from December 2015 through September 2018. The GIOCA is an academic multidisciplinary outpatient clinic in the Netherlands that specializes in fast-track diagnosis and treatment planning within a day [41], referred to as the *GIOCA day*. During the study, 691 patients visited the GIOCA; of these, 643 were successfully approached by telephone 1 to 5 days before their visit, depending on the day they received their referral. We informed patients about the purpose of the study (ie, to gain insight into information provision to patients with cancer) and offered them access to a website containing relevant information about GIOCA’s procedures, which could help them prepare for their visit. Of the 517 patients who had internet access and wanted to receive an email with access to the website, 241 consented to participate. As 9 of the included patients did not use the website, a total of 232 patients were included in the final analyses. Patients most often declined participation because they had no time or found it too burdensome. Only 8.0% (22/276) of declining patients explicitly mentioned that recording their consultation was a breach of their privacy, and only 8.0% (22/276) had no interested in additional (online) information. A nonresponse analysis revealed no differences between participating and nonparticipating patients in age, *t*_689_=1.52; *P*=.13, and gender, χ^2^_1_=3.2; *P*=.07. An overview of participant inclusion, reasons for nonresponse, randomization procedure, and dropout rates is presented in Figure 1.

### Website Intervention

A total of 4 website versions were developed containing the same information but presented in different modalities (via text, images, and/or patient videos). The nontailored website versions contained either text only, text with images, or text with videos. The text with videos version contained 6 videos featuring patients who narrated the textual information on the website (ranging from 1:30 to 3:00 min in length). As the images and videos were based on the textual content, they offered similar information. The information on the nontailored websites was offered in a standardized manner and could not be adapted by patients. The mode-tailored website version allowed patients to self-tailor the information presentation to their preferred mode at any moment during viewing. We did not include a nontailored version with all modalities, as we previously found that too much information on one Webpage can be detrimental for patient outcomes [18].

**Figure 2:**
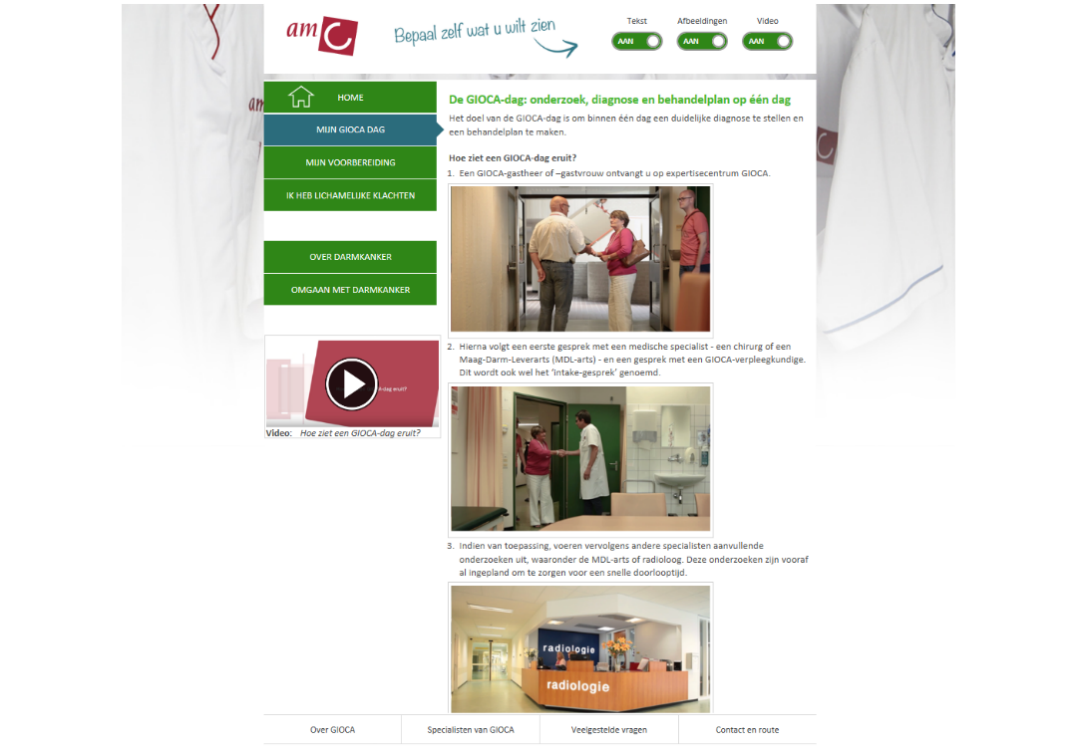
Example of the mode-tailored website with all modes switched on (text, images, videos).

**Figure 3:**
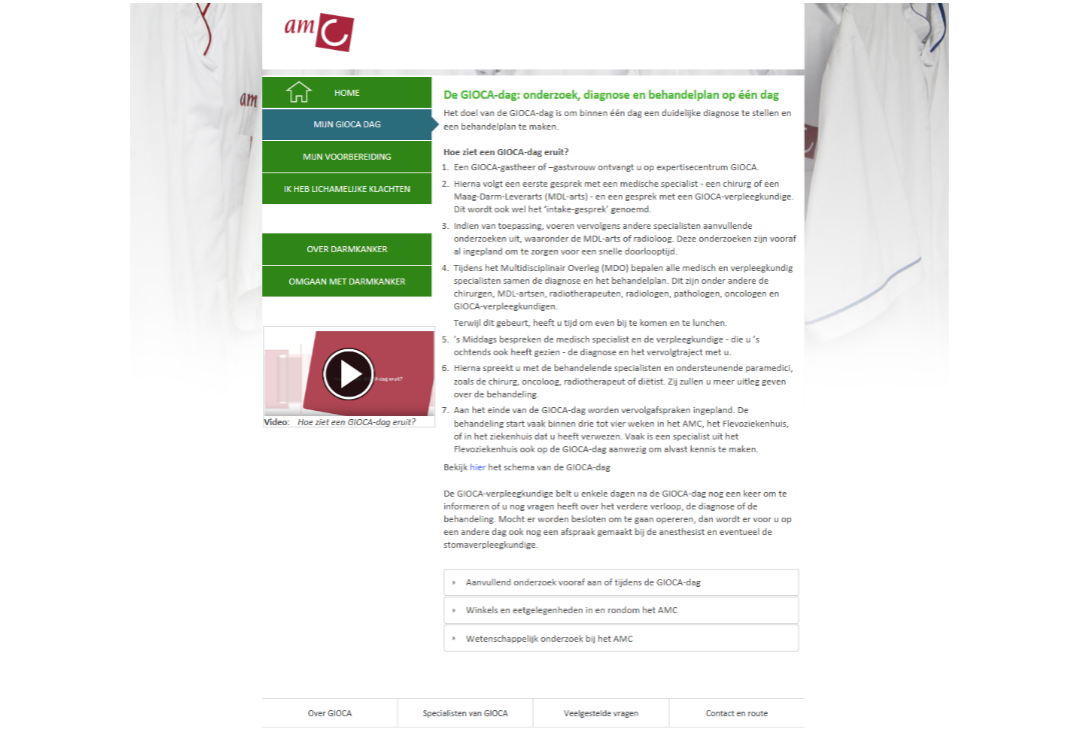
Example of a non-tailored website with text and video.

The website contained different pages with information about the fast-track clinic, how to prepare for consultations, and when to contact the clinic. Furthermore, the website contained information about the conditions (colorectal, stomach, or esophageal cancer), medical tests, treatment options, and practical information, such as a list of health care providers, frequently asked questions, and contact and location information. The content on the websites was similar for both patients with colorectal cancer and patients with stomach and esophageal cancer, except for the information concerning the condition and treatment options. Details on the development and content of these websites are published elsewhere [27]. Examples of the mode-tailored website and the nontailored websites are given in Figures 2 and 3. 

### Procedure

Consenting patients were stratified by age group and then randomized to one of the 4 conditions using randomization software. After the first telephone contact (1-5 days before visit), patients received a link to one of the 4 website versions by email from the study coordinator. Patients were not aware that there were other website versions than the one they received. Patients were free to use the website as they wished (how often, how long, and which pages). After viewing the website, consenting patients completed an online questionnaire to record website experience outcomes and background variables such as sociodemographic information, health background information, and information preference characteristics (T1a). One day before their visit to the clinic, patients’ knowledge acquired from the website was assessed by telephone (T1b). On the day of the patients’ visit to the clinic, a research assistant was present in the hospital to video record all consultations to assess question asking during consultations (T2). The fast-track program (GIOCA day) started with 2 intake consultations (medical specialist and nurse) to evaluate symptoms and medical history (T2). At noon, a multidisciplinary team discussed the diagnosis and formulated a treatment plan. In the afternoon, the diagnosis and treatment advice were discussed with the patient by the physician and nurse who conducted the intake consultations (T2). Depending on the treatment plan, patients also visited a surgeon, oncologist, or radiation oncologist on the same day to discuss treatment details (T2). Patients usually had 4 to 7 consultations during the GIOCA day, all of which were video recorded for this study. Immediately after the last consultation, a paper questionnaire was used to measure anxiety (T2). Patients were contacted by telephone within 36 to 48 hours after their visit to assess their knowledge from the website and information recall from the consultation (T3).

We took extensive measures to limit the overall participant burden. We had a trained research assistant who informed patients at the start of the study by telephone to ensure they knew exactly what the participation would entail, including how their privacy was safeguarded. Then, we sent out an information letter over email and contacted patients after 2 days again. Every patient was in contact with 1 research assistant who conducted telephone interviews before and after consultation and accompanied the patient in the hospital for data collection (handing out the questionnaires at the right time and installing the video recorder). This ensured that both patients and providers dealt with 1 person only and did not have to memorize when to fill out which questionnaire or turn on the video recording.

### Perceived Level of Tailoring of the Website

We measured to what extent patients felt the website was tailored to their situation with 2 items. The items comprised “the way I viewed information on the website (via text; text with images; text with video; text, images, and/or video) corresponded to my preference to receive health information” and “the presentation of the information on the GIOCA website was tailor-made for me.” The answer options ranged from 1 (totally disagree) to 7 (totally agree), with higher scores indicating higher levels of perceived tailoring (Pearson *r*=0.74; *P*<.001).

### Website Experience Outcomes (T1)

*Website use* patterns were recorded using a built-in Web tracker that logged every action on the website, including the number of clicks, time spent on each page, video viewing behavior, mode selections, and number of visits.

*Website involvement* was measured with 5 items, including “I was highly involved in evaluating the website” and “I carefully viewed the information on the website” [42]. Answer options ranged from 1 (totally disagree) to 7 (totally agree), of which mean scores were calculated (alpha=.81).

The 10-item version of the *Website Satisfaction Scale* [18,43] was used to assess the degree to which patients were satisfied with the (1) attractiveness of the website (3 items, eg, *the website looks nice*; alpha=.86), (2) comprehensibility of the website (3 items, eg, “the website is understandable”; alpha=.97), and emotional support from the website (4 items, eg “the website helps me with my emotions”; alpha=.92). Answer options ranged from 1 (totally disagree) to 7 (totally agree), of which mean scores were calculated.

*Communication self-efficacy* was measured with the short-form Perceived Efficacy in Patient-Physician Interactions questionnaire [44]. A total of 5 questions assessed the patient’s confidence in their ability to communicate with their provider on a scale from 1 (very confident) to 5 (not confident at all). A sum score was calculated (range 5-25; alpha=.88).

Patients’ current state of *anxiety* was measured with the 6-item version of the State-Trait Anxiety Inventory (STAI-6) [45]. Patients rated whether they experienced the presence (tense, upset, or worried) or absence (calm, relaxed, or content) of anxiety from 1 (not at all) to 4 (very much so). On the basis of the guidelines, the scores were recoded to range from 20 to 80, with scores >44 indicating high anxiety (alpha=.80) [46].

*Knowledge* acquired from the website was measured using the protocol of the Netherlands Patient Information Recall Questionnaire (NPIRQ) via telephone interviews [6,47]. We asked patients 12 standardized open questions based on the website content (eg, about the goal and course of the fast-track program and which medical specialists they will see). On the basis of a predeveloped code book, for 9 questions, patients could score 2 points, and for 3 questions, the correct answers contained fewer or more elements, and thus, these questions accounted for 1, 2.5, and 3 points. Thus, the maximum knowledge score at T1 for 12 questions (9×2 + 1×1 + 1×2.5 + 1×3) was 24.5. A standardized score was calculated by taking the percentage correctly answered according to the NPIRQ guidelines. The first author (MHN) coded all the answers. Additionally, answers from 14 patients were double coded by a second coder (FY), showing good intercoder reliability (mean kappa=0.737; *P*=<.001).

### Consultation Experience Outcomes (T2)

*Anxiety* immediately after consultation was measured in the same way as before the consultation (STAI-6; mean 41.45, *SD* 11.76; Cronbach alpha=.81).

To code the *question-asking behavior*, a codebook was developed based on earlier work by Zandbelt et al [48]. All questions during consultation related to (1) medical, (2) practical, and (3) paramedical information were coded. Questions on medical topics were about the patients’ disease, treatment (options), complications, and side effects. Questions on practical topics were about logistics of treatment and follow-up appointments. Questions on paramedical information were about psychosocial topics and consequences for daily life. Questions that were unrelated to the patient’s condition (eg, the weather and holiday) were not coded. All questions were summed into one total score. A total of 16 consultations were double coded, revealing good intercoder reliability (Krippendorff alpha=.951).

### Recall of Information and Knowledge After the Consultation (T3)

*Information recall* from the consultation and *knowledge* from the website were measured with the NPIRQ (similar to measurement of knowledge before the consultation; T1). Regarding *information recall* from the consultation, each participant was asked 13 standardized open questions (eg, about the proposed treatment plan, logistic planning of treatment, possible risks and side effects of treatment, and recommendations for daily life). To improve the validity of the recall measure, a maximum of 5 additional open questions were formulated, tailored to each patient’s videotaped consultations (eg, about details of treatment and additional medical tests). The correct answers were derived from the videotaped consultations. Each answer as provided by the participant during the interview was scored as not recalled (0), partially recalled (1), and completely recalled (2) based on a predeveloped code book. In theory, patients could receive a maximum of 18 questions (13 standardized + 5 tailored questions). However, as the content of consultations varied between patients, not all standardized open questions were applicable to all patients. Similar to T1, a standardized score was calculated by taking the percentage of correctly recalled information, based on the patient’s total sum score (1-23) and the maximum obtainable recall score (range 4-34; mean 56.37, *SD* 15.21).

The website contained information on 10 of the 13 standardized open questions asked. Hence, a separate *knowledge* score about topics on the website was calculated from these 10 questions. For this knowledge score, the website content was used as a guideline to score patients’ answers (similar to knowledge at T1). Again, a standardized score was calculated by taking the percentage correctly answered, based on the total sum score (0-9) and maximum obtainable score (range 10-19; *mean* 15.05, *SD* 11.76). The first author (MHN) coded all answers. A second coder (MA) coded answers from 14 participants from a different dataset with the exact same code book (mean kappa=0.816; *P*<.001) [6].

### Background Variables

#### Sociodemographic Information

Sociodemographic information included age, gender, and education level. Education level was divided into lower (ie, primary education, general secondary education, and middle vocational education) and higher education level (ie, higher vocational education and university).

#### Health Background Information

Health background information included cancer type (coded as colorectal cancer=0 and esophageal/stomach cancer=1) and whether patients came in for a second opinion (no=0 and yes=1). The treatment goal (palliative or unclear=0 and curative=1) was derived from the medical file. Health literacy was measured with the comprehension test of the Short Assessment of Health Literacy in Dutch that consists of 22 health-related words (eg, biopsy, ventricle, and palliative) [49]. For each word, people were asked to select the correct meaning out of 3 multiple choice options or an “I don’t know” option. The sum score of correct answers reflects their health literacy level and could range between 0 and 22. Patients’ frailty (ie, functioning in the physical, cognitive, social, and psychosocial domain) was assessed with the 15-item Groningen Frailty Indicator [50]. The quality of life was measured with 2 items from the European Organisation for Research and Treatment of Cancer Quality of Life Questionnaire [51] with answer options ranging from 1 (very bad) to 7 (excellent; Pearson *r=*0.75; *P*<.001).

#### Information-Seeking Characteristics

Internet use was measured in hours per week. Monitoring coping style refers to the degree to which patients seek information in a threatening medical situation. This was assessed with an adapted version of the Threatening Medical Situation Inventory (eg, “I intend to get as much information as possible about my treatment”) [24,52], using 3 items with answer options from 1 (not applicable to me at all) to 5 (very applicable to me; Cronbach alpha=.83). We assessed information preference with an adapted item from the Information Satisfaction Questionnaire [53], asking whether patients prefer to receive (1) “as much information as possible, both positive and negative,” (2) “as much information as possible, both positive and negative, but bit by bit,” (3) “not much information,” and (4) “only positive information”. In conformity with previous research [54], the items were dichotomized by merging category (2), (3), and (4) into “not all information (at once)” (0) versus “as much information as possible, both positive and negative” (1). Finally, we assessed whether patients received information about the clinic from other sources (eg, health care providers and brochures) besides our website (no=0 and yes=1).

### Statistical Analyses

Chi-square tests, *t* tests, and ANOVAs were conducted to check for unequal distribution of background variables over conditions. Descriptive analyses were used to explore patterns of website use. Main and interaction effects of mode tailoring (H1-H6, RQ1-2) were tested with ANOVAs. Additional simple effects analyses were conducted to examine differences between conditions within age groups. The significance level was set at *P*<.05. To test which website experience outcomes predicted knowledge before the consultation (T1) and how these, together with consultation experience outcomes (T2), predicted information recall from the consultation and knowledge from the website after the consultation (T3; RQ3-4), 3 multistage linear regression models were estimated. All analyses started with a baseline model (Model 1) of individual background variables (with age as a continuous variable). Website experience outcomes were added as predictors in Model 2. For information recall from the consultation and knowledge from the website at T3, relevant consultation experience outcomes (T2) were included as predictors in Model 3. To reduce the number of predictors, only variables that were at least marginally correlated with knowledge or information recall were included in the models (*P*<.10). Assumptions of linearity, normality, homoscedasticity, independent errors (Durbin-Watson values for Model 1, 2, and 3 are 1.95, 1.89, and 1.75, respectively) and multicollinearity (variance inflation factor<10) were met for all variables. Standardized coefficients (betas) are reported for comparisons of predictive power.

## Results

### Patient Characteristics

Participating patients were aged, on average, 63.50 years (*SD* 9.06; range 36-86), with 46.1% (107/232) aged ≥65 years. The majority were male (68.1%, 158/232) and lived together with their spouse, children, or other family members (82.8%, 192/232). The majority were advised a curative treatment plan (73.2%, 170/232), whereas 13.4% (31/232) entered a palliative trajectory, and 13.4% (31/232) were scheduled for additional imaging studies to formulate a clear diagnosis and treatment plan. Of the 232 participating patients, 74 viewed the mode-tailored website, 53 viewed the text-only website, 54 viewed the text with images website, and 51 viewed the text with video website. Background information of the patients is given in Table 1.

**Table 1:** Patient background characteristics.

Background variables^a^			Older patients (n=107)	Younger patients (n=125)	All patients (n=232)	Total (N)^b^
**Sociodemographic information**						
	Age (years), mean (SD)		71.44 (4.23)	56.81 (6.18)	63.50 (9.06)	232
	**Gender**					
		Male, n (%)	77 (72.6)	81 (64.3)	158 (68.1)	232
		Female, n (%)	29 (27.4)	45 (35.7)	74 (31.9)	232
	**Education level**					
		Lower, n (%)	71 (67.0)	75 (59.5)	146 (62.9)	231
		Higher, n (%)	35 (33.0)	50 (39.7)	85 (37.1)	231
**Health background information**						
	**Cancer type** ^c^					
		Colorectal, n (%)	77 (72.6)	110 (87.3)	187 (77.9)	232
		Esophageal/stomach, n (%)	29 (27.4)	16 (12.7)	45 (22.1)	232
	**Second opinion**					
		No, n (%)	90 (84.9)	99 (78.6)	189 (81.5)	232
		Yes, n (%)	16 (15.1)	27 (21.4)	43 (18.5)	232
	**Treatment goal**					
		Palliative, n (%)	13 (12.3)	18 (14.3)	31 (13.4)	232
		Curative, n (%)	81 (76.4)	89 (70.6)	170 (73.2)	232
		Unclear, n (%)	12 (11.3)	19 (15.1)	31 (13.4)	232
	Health literacy^d^, mean (SD)		16.50 (4.99)	16.82 (4.38)	16.66 (4.66)	182
	Frailty^e^, mean (SD)		2.19 (1.86)	2.69 (2.05)	2.46 (1.98)	182
	Quality of life^f^, mean (SD)		5.24 (1.17)	4.99 (1.36)	5.11 (1.28)	229
**Information characteristics**						
	Internet useb^b,g^, mean (SD)		12.02 (10.30)	17.98 (17.74)	15.27 (15.10)	229
	Information coping style^h^, mean (SD)		3.74 (0.87)	3.76 (0.87)	3.75 (0.87)	229
	**Information preference**					
		Not all information, n (%)	25 (24.0)	31 (24.8)	56 (24.5)	229
		As much information as possible, n (%)	79 (76.0)	94 (75.2)	173 (75.5)	229
	**Additional information received**					
		No, n (%)	30 (30.9)	38 (31.4)	68 (31.2)	218
		Yes, n (%)	67 (69.1)	83 (68.6)	150 (68.8)	218

^a^No differences were found between conditions.

^b^*N* refers to the entire population under study and *n* refers to a sample population under study. Not all cells add up to 100% owing to missing data.

^c^Differs significantly between younger and older patients at *P*<.01.

^d^A higher score indicates higher levels of health literacy (maximum range: 0-22; reported range 0-22).

^e^A higher score indicates higher frailty (maximum range 1-15; reported range 0-10).

^f^A higher score indicates higher quality of life (maximum range 1-7; reported range 2-7).

^g^Measured in hours per week.

^h^A higher score indicates a higher information monitoring coping style (maximum range 1-5; reported range 1-5).

### Perceived Level of Tailoring

Patients viewing the mode-tailored website version had equally high perceptions of the degree to which the information presentation was tailored to them as compared with those viewing the nontailored versions (*mean* 5.24, *SD* 1.22; *F*_3,225_=0.19; *P=*.91; ηp^2^=0.00).

### Website Use Patterns

Of the 232 participating patients, 74 viewed the mode-tailored website (31.9%), 53 viewed the text-only website (22.8%), 54 viewed the text with images website (23.3%), and 51 viewed the text with video website (23.0%). Patients spent an average of 34 min and 45 seconds on the website (*SD* 00:32:56; range 00:00:34-03:50:42). Patients who received the website a day before their visit did not spend less time on the website than those who received it earlier (*t*_230_=1.79; *P*=.07) and this did not differ between conditions (χ^2^_3_=1.1; *P*=.76). The majority of patients (62.1%, 144/232) visited the website twice or more in the days before their visit (mean 2.78, *SD* 2.28; range 1-22). Patients mostly consulted information about the GIOCA day (90.9%, 211/232), how to prepare for their visit (86.6%, 201/232), their condition (colorectal, stomach, or esophageal cancer; 80.6%, 187/232), and how to deal with cancer in daily life (ie, nutrition, fatigue, and psychosocial care; 67.2%, 156/232), and with which symptoms to contact the hospital (65.1%, 151/232). Almost half of the patients viewed information on diagnostic tests (40.5%, 94/232) and frequently asked questions (48.3%, 112/232). Contact information (27.2%, 63/232), information about which medical specialists work at GIOCA (19.8%, 46/232), and information on additional websites (25.0%, 58/232) were least often consulted. An overview of website use patterns is presented in Table 2.

Videos were available for patients in the text with video condition and mode-tailored condition (total *n*=125). Of these patients, 41 (32.8%; *n*_tailored_=18; *n*_video_=23) watched a total of 96 videos on the website. Within the conditions, 28% (21/74) of patients in the tailored condition watched a video compared with 39% (20/51) in the text with video condition. Within age groups, 29% (16/54) of older patients watched a video compared with 35% (25/71) of younger patients. These differences were not significant. Most patients who watched a video, watched it almost completely (total video time: mean 00:06:52, *SD* 00:05:19, range 00:15-22:35). The majority of patients who watched videos, watched more than one (61%; 25/41).

Patients in the mode-tailored condition spent an average of 43:07 min on the website, compared with 30:59 min for patients in the text condition, 33:52 min for patients in the text with images condition, and 26:26 min for patients in the text with video condition. However, this difference was not significant (*F*_3,224_=2.52; *P*=.06; ηp^2^=0.03). There were no differences between age groups in terms of time spent on the website (*F*_3,224_=0.00; *P*=.96; ηp^2^=0.00). All patients in the mode-tailored condition chose at least text, but the majority supplemented this with additional images or videos spread over multiple visits to the website. The majority of patients (77%, 57/74) in the mode-tailored condition selected all 3 modalities (text, images, and video); 16% of patients (12/74) chose text and images; 1% of patients (4/74) chose text only; and only 1/74 patient chose text with video. Regarding the first time on the website, most patients first chose text (79%, 59/74); 13% of patients first chose images (10/74); and 6% of patients first chose video (5/74). During subsequent Web sessions, patients were more likely to choose images and video first. On average, patients took 01:15 min to select their first mode (*SD* 02:10; 39%, 29/74 <30 seconds, 66%, 50/74 <1 min, and 86.5%, 64/74 <2 min). Regarding the second visit, the first mode was selected on average at 29 seconds (*SD* 00:51; 84%, 32/38 <30 seconds, 94.7%, 36/38 <2 min).

**Table 2:** Patterns of website use.

Website use variables		Older patients (n=106)	Younger patients (n=126)	All patients (n=232)
**Time spent on website (mm:ss), mean (SD)**		34:27 (32:09)	35:00 (33:42)	34:45 (32:56)
	Mode-tailored (31.9%, n=74)	41:35 (40:55)	44:14 (43:01)	43:07 (41:53)
	Text-only (22.8%, n=53)	30:25 (39:34)	31:31 (22:11)	30:59 (25:49)
	Text with images (23.3%, n=54)	34:09 (30:34)	33:35 (35:23)	33:52 (32:51)
	Text with video (23.0%, n=51)	29:42 (21:55)	25:33 (20:15)	26:26 (20:55)
**Web pages, n (%)**				
	The GIOCA^a^-day	90 (84.9)	121 (96)	211 (90.9)
	Preparing for the GIOCA-day	84 (79.2)	117 (92.9)	201 (86.6)
	Information about cancer types	84 (79.2)	103 (81.7)	187 (80.6)
	Diagnostic tests	49 (46.2)	45 (35.7)	94 (40.5)
	When to contact the hospital	62 (58.5)	89 (70.6)	151 (65.1)
	Daily life recommendations	65 (61.3)	91 (72.2)	156 (67.2)
	Additional relevant websites	23 (21.7)	35 (27.8)	58 (25.0)
	Frequently asked questions	48 (45.3)	64 (50.8)	112 (48.3)
	Medical specialists at GIOCA	23 (21.7)	23 (18.3)	46 (19.8)
	Contact information	31 (29.2)	32 (25.4)	63 (27.2)
**Watched at least one video^b^, n (%)**		16 (29.6)	25 (35.2)	41 (32.8)
	Mode-tailored	9 (29.0)	12 (27.9)	21 (28.4)
	Text with video	7 (30.4)	13 (46.4)	20 (39.2)
**Number of videos watched^c^, n (%)**		2.50^d^ (2.50)	2.24^e^ (1.30)	2.34^d^ (1.84)
	Mode-tailored	3.00^d^ (3.28)	2.50^e^ (1.57)	2.71^d^ (2.39)
	Text with video	1.86^f^ (0.69)	2.00^g^ (1.00)	1.95^g^ (0.89)
**Number of mode actions^h^, mean (SD)**		2.97 (7.27)	2.72 (6.26)	2.84 (6.72)
**Time until first mode (mm:ss)^h^, mean (SD)**		01:00 (00:48)	01:25 (02:45)	01:15 (02:10)
	First mode ≤1 min (%)	64.5	67.4	66.2
	First mode ≤2 min (%)	87.1	86	86.5
	First mode ≤4 min (%)	100	95.3	97.3
**First mode chosen^b,h^, n (%)**				
	Text	27 (87.1)	32 (74.4)	59 (79.7)
	Illustrations	4 (12.9)	6 (14.0)	10 (13.5)
	Video	0 (0.0)	5 (11.6)	5 (6.8)
**Mode combinations^b,h^, n (%)**				
	All 3 modes	23 (74.2)	34 (79.1)	57 (77.0)
	Text and illustrations	8 (25.8)	4 (9.3)	12 (16.2)
	Text and video	0 (0.0)	1 (2.3)	1 (1.4)
	Text only	0 (0.0)	4 (9.3)	4 (5.4)

^a^GIOCA: Gastro-Intestinal Oncological Centre Amsterdam

^b^Only applicable to patients viewing the mode-tailored (n=74) and text with video website (n=51).

^c^Only includes patients who watched at least one video.

^d^Range: 1-11.

^e^Range: 1-6.

^f^Range: 1-3.

^g^Range: 1-4.

^h^Only applicable to patients viewing the mode-tailored website (n=74).

### Effects on Website Experience Outcomes Before Consultation (T1)

Table 3 shows the summary statistics of all outcomes. We hypothesized that exposure to a mode-tailored website (vs nontailored websites) would positively affect patients’ website involvement, website satisfaction, anxiety, communication self-efficacy, and knowledge before the consultation (H1). Our data showed no significant differences between the conditions for these website experience outcomes. We also hypothesized differential effects of mode tailoring for younger and older patients, with stronger effects for older patients (H2). Although no significant interaction effects were present, a simple effects analysis revealed that, in contrast with our hypothesis, for younger patients the mode-tailored website (mean 5.12, *SD* 0.97; *P*=.02) and text with images website (mean 5.30, *SD* 0.91; *P*=.009) resulted in higher satisfaction with the attractiveness of the website compared with the text-only website (mean 4.46, *SD* 1.08). In general, older patients had lower knowledge levels (mean 22.70, *SD* 12.57) than younger patients (mean 30.16, *SD* 13.00; *F*_3,218_=17.91; *P*<.001; ηp^2^=0.08). Overall, the data showed no support for H1 and H2.

**Table 3:** Means and standard deviations of patient outcome variables.

Patient outcome variables		Mode-tailored, mean (SD)		Text-only, mean (SD)		Text with images, mean (SD)		Text with video, mean (SD)		Total, mean (SD)	
		Young	Old	Young	Old	Young	Old	Young	Old	Young	Old

**T1: Website experience outcomes**											
	Website involvement	4.9 (11.0)	4.7 (1.1)	4.5 (1.0)	4.7 (1.21)	4.8 (1.1)	4.6 (1.3)	4.5 (1.1)	4.9 (1.0)	4.7 (1.1)	4.7 (1.1)
	Website attractiveness	5.1^a,b^ (1.0)	5.1 (1.2)	4.5 (1.1)	5.2 (1.4)	5.3^a,c^ (0.9)	4.9 (1.33)	4.9 (1.2)	4.9 (1.3)	5.0 (1.1)	5.0 (1.3)
	Website comprehension	6.4 (1.4)	6.2 (0.8)	6.2 (0.7)	6.0 (1.2)	6.6^b,d^ (0.5)	6.0 (1.3)	6.0 (1.4)	5.8 (1.4)	6.3 (0.9)	6.0 (1.2)
	Website emotional support	3.9 (1.3)	4.1 (1.2)	3.7 (1.2)	4.1 (1.7)	4.1 (1.3)	3.9 (1.4)	3.8 (1.4)	4.2 (1.5)	3.9 (1.3)	4.1 (1.4)
	Self-efficacy	20.4 (2.5)	20.0 (3.4)	19.7 (3.8)	20.7 (3.0)	20.2 (2.7)	21.2 (3.00)	21.4^a,b^ (3.4)	20.0 (3.1)	20.4 (3.1)	20.5 (3.2)
	Anxiety	48.4 (11.2)	48.1 (11.1)	48.5 (10.1)	43.6 (11.0)	48.0 (11.2)	48.6 (8.4)	45.8 (10.9)	45.5 (10.0)	47.8 (10.8)	46.5 (10.3)
	Knowledge	32.1 (12.2)	25.4 (13.4)	32.7 (13.5)	20.8 (11.6)	27.6 (11.1)	21.0 (14.1)	27.4 (15.0)	23.2 (10.4)	30.2 (13.0)	22.7^e,f^ (12.6)
**T2: Consultation experience outcomes**											
	Question asking	24.8 (21.3)	19.7 (14.9)	19.8 (13.1)	14.9 (11.9)	17.3 (11.6)	17.4 (23.2)	19.7 (17.0)	15.6 (11.2)	20.9 (17.0)	17.1 (15.9)
	Anxiety	39.6^a,b^ (11.0)	44.1^a,b^ (12.6)	45.8 (12.8)	37.4 (10.9)	44.9 (12.0)	40.1 (12.4)	41.8 (12.1)	38.6 (9.9)	42.5 (12.0)	40.3 (11.7)
**T3: Outcomes after consultation**											
	Knowledge from website	19.1 (12.1)	12.4 (10.8)	21.2 (13.4)	12.9 (10.9)	13.1^a,b^ (11.2)	11.1 (9.4)	17.4 (15.3)	10.4 (8.3)	18.0 (13.1)	11.8^e,f^ (9.9)
	Information recall consultation	57.4 (11.4)	56.2 (15.2)	51.9 (13.4)	55.2 (18.8)	57.3 (16.6)	55.4 (15.5)	62.6^b,d^ (15.2)	54.3 (17.1)	57.3 (14.2)	55.4 (16.3)

^a^Differs from text-only condition.

^b^ Significant at *P*<.05.

^c^ Significant at *P*<.10.

^d^Differs from text with video condition.

^e^Differs from younger patients.

^f^Significant at *P*<.001.

### Effects on Consultation Experience Outcomes (T2)

We explored whether exposure to a mode-tailored website would influence the number of questions asked by patients during consultations (RQ1) and whether this would differ between younger and older patients (RQ2). Results showed no significant differences between condition and no interaction effects between condition and age for the number of questions asked during consultations. Additionally, we hypothesized that exposure to a mode-tailored website (vs nontailored websites) would decrease anxiety immediately after consultation (H3), with stronger effects for older patients (H4). Although we found no main effect of condition, a significant interaction between condition and age group was revealed (*F*_3,204_=3.16; *P=*.03; ηp^2^=0.04). However, in contrast with our expectations, older patients reported higher anxiety in the mode-tailored condition (*mean* 44.14, *SD* 12.62; *P*=.04) compared with the text condition (*mean* 37.36, *SD* 10.86). On the contrary, younger patients reported lower levels of anxiety in the mode-tailored condition (*mean* 39.59, *SD* 10.97; *P=*.046) compared with the text condition (*mean* 45.80, *SD* 12.80). Overall, the data showed no support for H3 and contrasting results for H4.

### Effects on Information Recall and Knowledge After Consultation (T3)

We hypothesized that exposure to a mode-tailored (vs nontailored) website would increase patients’ knowledge from the website and information recall from the consultation (H5), with differential effects for younger and older patients (H6). For both outcomes, we found no significant differences between conditions and no interaction effects. Overall, older patients acquired less knowledge from the website (*mean* 11.78, *SD* 9.88) than younger patients (*mean* 17.96, *SD* 13.12; *F*_1,194_=12.89; *P*<.001; ηp^2^=0.06). There were no age differences in recall from the consultation (*mean*_younger_=57.29, *SD* 14.15; *mean*_older_=55.35, *SD* 16.33).

### What Motivation- and Ability-Related Factors Explain Knowledge and Information Recall Before and After Consultation?

Table 4 summarizes all regression models. Regarding knowledge from the website before the consultation (T1), the baseline model with individual background variables (Model 1; n=211) revealed that younger age (beta=–.23; *P*=.001) and higher education levels (beta=.22; *P*=.002) were associated with higher knowledge. Patients who received information about the clinic from other sources also reported higher knowledge (beta=.13; *P*=.06); however, this effect was not significant. Extending this model with website experience outcomes (Model 2) significantly improved the model (∆*R*²=0.06; *P*=.006; total adjusted *R*²=0.17). Higher perceived website involvement (beta=.15, *P=*.03) and higher satisfaction with the comprehensibility of the website (beta=.15, *P=*.05), although the latter borderline significant, are associated with higher knowledge at T1.

Regarding information recall from the consultation (T3), no background variables were associated with information recall (Model 1, n=194). Extending the model with website experience outcomes revealed that knowledge before the consultation (beta=.22; *P*=.003), whether the patient had watched a video on the website (beta=.14; *P*=.07), and communication self-efficacy (beta=.12; *P*=.09) explained a significant additional proportion of variance in information recall (∆*R*²=0.12; *P*=.001; total adjusted *R*²=0.09). The latter 2 variables were however not significantly related to information recall. No consultation experience outcomes were associated with information recall from the consultation at T3.

Regarding knowledge from the website after the consultation (T3), the baseline model with control variables (Model 1, n=185) revealed that younger age (beta=–.18; *P*=.02) and higher education levels (beta=.16; *P*=.03) were associated with higher knowledge. Extending this model with website experience outcomes significantly improved the model (∆*R*²=0.19; *P*<.001; total adjusted *R*²=0.27). Specifically, more time spent on the website before the consultation (beta=.21; *P*=.002) and higher knowledge at T1 (beta=.39; *P*<.001) were associated with higher knowledge at T3. Age (beta=–.08; *P=*.23) and education level (beta=.10; *P*=.12) became insignificant predictors of knowledge. No consultation experience outcomes were associated with knowledge at T3.

**Table 4:** Regression models predicting knowledge and information recall.

Regression outcomes^a^		Website knowledge (T1; n=211)^b^				Information recall consultation (T3; n=194)^c^				Website knowledge (T3; n=185)^d^					
		Model 1		Model 2		Model 1		Model 2		Model 1		Model 2		Model 3	
		Beta	*P* value	Beta	*P* value	Beta	*P* value	Beta	*P* value	Beta	*P* value	Beta	*P* value	Beta	*P* value
**Individual background characteristics**															
	Age (years)	-0.23	.001	-0.23	.001	-0.08	.26	-0.04	.59	-0.18	.02	-0.08	.23	-0.08	.24
	High education level	0.22	.002	0.22	.002	-0.02	.84	-0.05	.51	0.16	.03	0.1	.12	0.1	.14
	Internet use	-0.02	.75	-0.02	.72	—^e^	—	—	—	0.09	.22	0.05	.48	0.05	.49
	Coping style	0.11	.11	0.04	.61	—	—	—	—	—	—	—	—	—	—
	Additional information received	0.13	.05	0.1	.11	—	—	—	—	—	—	—	—	—	—
	Quality of life	—	—	—	—	—	—	—	—	-0.13	.08	-0.1	.15	-0.1	.16
**Website experience characteristics**															
	Website involvement	—	—	0.15	.03	—	—	—	—	—	—	—	—	—	—
	Website attractiveness	—	—	-0.01	.87	—	—	0.08	.32	—	—	—	—	—	—
	Website comprehension	—	—	0.15	.05	—	—	-0.02	.78	—	—	—	—	—	—
	Watched a video	—	—	0.08	.22	—	—	0.14	.07	—	—	—	—	—	—
	Knowledge (T1)	—	—	—	—	—	—	0.22	.003	—	—	0.39	.001	0.39	.001
	Website emotional support	—	—	—	—	—	—	0.07	.36	—	—	—	—	—	—
	Communication self-efficacy	—	—	—	—	—	—	0.12	.09	—	—	—	—	—	—
	Time on website	—	—	—	—	—	—	0.07	.36	—	—	0.21	.02	0.21	.002
	Anxiety (T1)	—	—	—	—	—	—	—	—	—	—	0.04	.52	0.04	.52
**Consultation experience characteristics**															
	Question asking	—	—	—	—	—	—	—	—	—	—	—	—	0	.96

^a^Only variables marginally significant (*P<*.10) that correlated with the predicted outcome variable were included. As no consultation characteristics correlated with information recall from the consultation (T3), only 2 models were predicted. Model 1 shows a simple linear regression model assessing the relationship between control variables and knowledge/information recall. Website experience characteristics were added to Model 2. Consultation experience was included in Model 3. We report the models without controlling for health literacy owing to missing data. Repeating the analyses with health literacy in the models did not change results, although health literacy significantly related to website knowledge (T3). *R*² indicates the adjusted explained variance of the model; ∆*R*² shows the change in *R*² by adding predictors in Model 2 and 3; significant ∆ *F* shows whether the difference in the *F* value for model expansion is significant.

^b^Adjusted *R*^2^ Model 1=0.12, Model 2=0.17). Adding website experience characteristics to Model 2 improved the model (∆*R*²=0.06, *P*=.006).

^c^Adjusted *R*^2^ Model 1=0.00, Model 2=0.09). Adding website experience characteristics to Model 2 improved the model (∆*R*²=0.12, *P*=.001).

^d^Adjusted *R*^2^ Model 1=0.09, Model 2=0.27, Model 3=0.27). Adding website experience characteristics to Model 2 improved the model (∆*R*²=0.19, *P*<.001). Addition consultation experience characteristics to Model 3 did not improve the model (∆*R*²=0.00, *P*=.96).

^e^Not applicable.

## Discussion

### Principal Findings

This RCT tested the effectiveness of a mode-tailored preparatory website (ie, by self-selecting text, images, and/or videos) versus nontailored websites (ie, with either text only, text with images, or text with videos) in a clinical population of older (≥65 years) and younger (<65) patients visiting a fast-track clinic for diagnosis and treatment planning for colorectal, esophageal, or stomach cancer. The main research question was whether mode tailoring is more effective than nontailored information on patient-reported outcomes before, during, and after consultation. Moreover, we investigated whether older patients benefited proportionally more from mode-tailored information than younger patients. To advance theoretical models on information processing and the interplay between online information provision and offline patient-provider communication, we additionally explored which *website experience outcomes* and *consultation experience outcomes* contributed to patients’ knowledge acquisition from online health information and information recall from consultations. Following is a review of the study results in light of the unexpected findings, discuss the implications for theory, and suggest directions for future research.

### Review of Findings

The first main and unexpected finding of this study was that younger patients were more satisfied with the mode-tailored website (vs text only), whereas this was not the case for older patients. Moreover, younger patients who viewed the mode-tailored website reported lower anxiety levels immediately after consultation (vs text only). In contrast with our hypothesis, older patients reported higher anxiety in the mode-tailored condition (vs text only). Posthoc analyses revealed that younger patients also showed greater anxiety *reduction* from pre- to postconsultation, after viewing the mode-tailored website (vs the nontailored websites), whereas the anxiety levels of older patients in the mode-tailored condition remained the same. Both younger and older patients had equal anxiety levels before consultation. On the basis of the socioemotional selectivity theory, we expected that older patients would perceive more emotional gratification if they had the option to select information presented in visual/audiovisual modes, thereby limiting their anxiety. Alternatively, we now discuss a different explanation for our findings, from an uncertainty management theory perspective combined with the socioemotional selectivity theory. Generally speaking, younger adults are less tolerant to uncertainty than older adults [55] and, therefore, more likely to seek information as a strategy to reduce uncertainty [56,57]. It could be that, in our study, younger patients were more intolerant to the uncertainty that came with their cancer diagnosis and, therefore, exhausted all their information sources (ie, different information modes) to reduce uncertainty and, thereby, their anxiety. Older adults, on the contrary, are generally better at tolerating and managing uncertainty [55] and could be less urged to reduce it by means of information. Information might even have reversed effects and increase anxiety in this group, especially among *older-old* patients with cancer (≥70 years), who more often prefer to leave information disclosure up to the health care provider [58]. Even though younger and older patients did not differ in information seeking/avoidance in this study, age-related differences in uncertainty tolerance might explain why providing information in tailored multiple modes particularly benefited younger patients, while it had no effect on older patients. The socioemotional selectivity theory also presents an explanation for this finding. Namely, younger adults pertain more to knowledge-related goals to prepare for future events, whereas older adults attach greater importance to emotionally meaningful goals [33]. It is possible that being able to view information in different (visual) modalities in a tailored manner accommodated to younger patients’ information needs (ie, knowledge-related goals) and supported them in lowering their anxiety, which was less so for older patients. Future research is warranted to understand the role of uncertainty intolerance and information seeking/avoidance, as well as knowledge- versus emotional-related goals, in elucidating whether and how online tailored health information might accommodate the needs of and benefit younger and older patients with cancer.

Overall, we did not find that mode tailoring proportionally benefitted older patients more than younger patients. Possibly, older patients had more difficulty using the mode tailoring functionality, whereas this was more intuitive for younger patients, because of differences in internet experience (see Table 1). More long-term use and experience with the tailoring tool could make the mode-tailored website become beneficial for older patients as well. Alternatively, in our study sample, many patients were in their 60s (41.8%). Despite the significant age difference of younger and older patients, many patients were aged around the cut-off of 65 years. This could explain why the age groups did not differ on age-related background variables and why no age differences were found in outcome variables. To illustrate, older patients in this study were not frailer than younger patients. In fact, the mean frailty score was 2.46 (*SD* 1.98), with less than a quarter of patients (23.6%) reporting a score higher than 4, a cut-off used to identify patients as (moderately) frail [49]. Recent work found that age-related factors (eg, frailty, health literacy, and future time perspective) are more predictive of information recall from cancer websites than chronological age [40]. Moreover, older adults are a highly heterogeneous group in fundamental domains such as biological, cognitive, and personality characteristics [59], which could influence how online health information is used, processed, and evaluated [60]. Therefore, when investigating website use behaviors and intervention effects on patient outcomes, it might be meaningful to consider age-related variables as moderators of effects.

The second main finding of this study is that certain website experience outcomes (eg, website involvement and time spent on the website) increased patients’ knowledge before and after the consultation. Higher previsit knowledge in turn supports patients in recalling information from the consultation. This suggests that offering information to help patients prepare for their hospital visit can improve past knowledge, which is important for how they process information during consultations, and remember this afterward. This is an important outcome, as knowledge is one of the key prerequisites for patients to be able to be involved in making treatment decisions and manage their illness [61]. Moreover, the website was widely used by patients across all website conditions (*mean* 34 min), and this was not affected by age, gender, or education level. The majority of patients even used the website multiple times before their hospital visit. This underlines a desire for information before a hospital visit, perhaps even more so in emotionally charged contexts such as the diagnosis and treatment planning phase. What makes these findings particularly noteworthy is that in this study, we did not find a relationship between anxiety during consultation and information recall from the consultation. In a previous observational field study among the same patient population, at the same outpatient clinic, but without a preparatory website intervention, anxiety during consultation negatively predicted information recall from consultations [6]. Interestingly, in this RCT, anxiety was not found to be a barrier for information recall. A comparison between the 2 samples reveals that patients in both studies reported equally high anxiety levels. This raises the question: Could it be that offering patients’ preparatory online information before their hospital visit helped them to attend to and process information from the consultation despite their anxiety levels? Previous studies showed that highly anxious patients with cancer have higher information needs [62] and that patients with fulfilled information needs are less anxious [63]. These findings, together with the results from our previous observational study and this RCT, suggest that knowledge (ie, by means of online health information) may play an important role in patients’ anxiety management overall, especially for younger patients. However, to answer this question with more accuracy, future research is warranted to understand the added value of offering online preparatory information (vs no information) on patients’ fulfillment of information needs, knowledge/information recall, and the anxiety-recall relationship.

Although no differences were found on main outcome variables, our data showed that patients spent more time on the mode-tailored website than on the nontailored website versions. This difference was not significant, but it suggests that mode tailoring may trigger patients to attend to the website information longer, which in this study proved to be important for knowledge acquisition from the website and information recall from consultations. The results align with earlier experimental findings that mode tailoring online information can increase attention to website information and consequently enhance information recall [17]. Although it is possible that the extra time spent on the mode-tailored website was because patients needed more time to figure out how the mode tailoring tool worked, it is more likely that patients spent this time viewing the website content. Namely, the time range that patients needed to select their first mode does not weigh up to the extra time patients spent on the mode-tailored website (vs the nontailored websites). Moreover, posthoc analyses revealed that viewing the mode-tailored website required comparable levels of cognitive effort as the nontailored versions for both younger and older patients (mean 2.65, *SD* 1.05; range 1-7 with higher scores indicating higher cognitive load, *F*_3,229_=0.23; *P*=.88; ηp^2^=0.00). In addition, even though no clear differences were found between conditions on the hypothesized outcome variables, the mode-tailored website kept patients online for a slightly longer period of time, which is likely to be important for knowledge gain. Together, the results suggest that offering online information to patients as a preparation tool might benefit patient outcomes.

### Theoretical Implications

**Figure 4:**
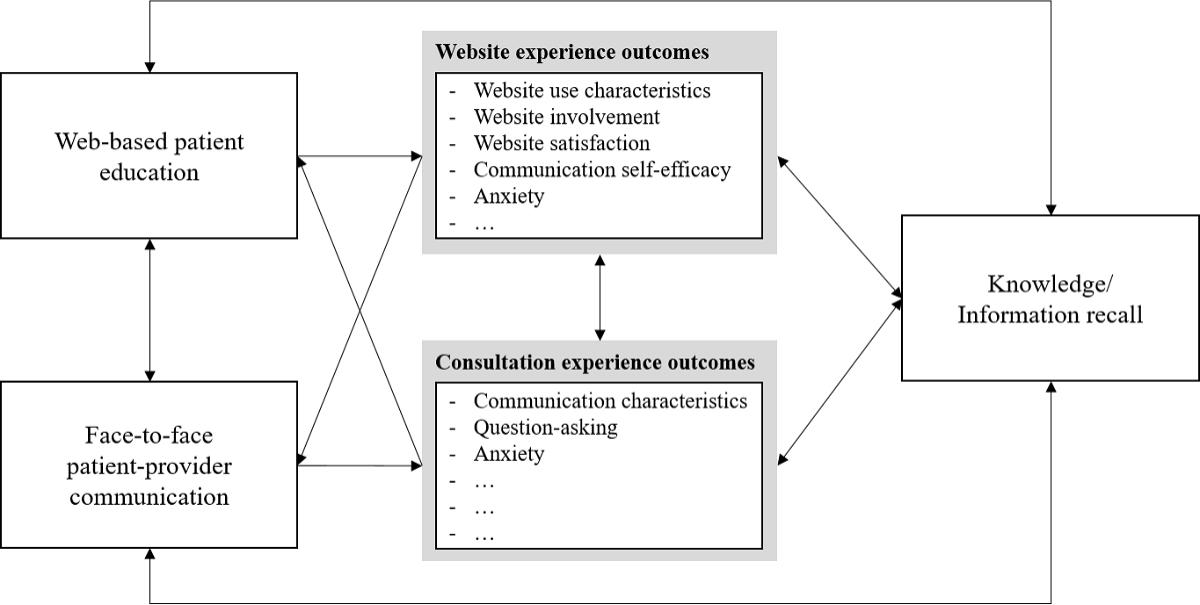
Integrated model of website experience outcomes and consultation experience outcomes explaining knowledge/information recall.

This study aimed to gain insight into which *website experience outcomes* and *consultation experience outcomes* explain the benefits of preparatory online information on knowledge acquisition from websites and information recall from consultations in the cancer communication context. As such, the results of this study are helpful in refining existing theoretical frameworks that conceptualize the interplay between mediated health communication and offline patient-provider communication in an eHealth era. At present, new information technologies open up a wide range of possibilities for patients to obtain health information outside of the consultation room. Scholars have conceptualized models of patient-provider communication that consider the role of mediated health communication to explain how communication can affect health outcomes via different pathways, such as by increasing patient knowledge, enhancing patients’ ability to manage emotions, and enhancing patient empowerment and agency [64,65]. Although such overviews are a useful starting point, our findings help further specify which specific pathways, considering both online information provision and offline patient-provider communication, are key in improving patient health outcomes. Specifically, we identified variables related to attention (ie, time spent online) and website involvement as important *motivational website experience* *outcomes* that contribute to processing of online information, thereby increasing patients’ knowledge. Although not one of the *consultation experience outcomes* (ie, question asking and anxiety) was related to information recall from consultations, we identified increased knowledge levels before consultation as an *ability-related website experience outcome* that benefitted patients’ recall of information from the consultations. These results also inform future research relying on theories such as the ELM and LC4MP to understand which specific motivation- and ability-related factors play a role in how online cancer information is processed and how this may impact patient outcomes. This is important, as a critique on the ELM has been that its mediating variables are not clearly defined, leaving room for further specification of which variables contribute to motivation and ability to process information in different situational contexts [66]. We conclude with an integrated model visualizing how the use of online health information and patient-provider communication may lead to website experience outcomes and consultation experience outcomes, which may reinforce each other and ultimately explain knowledge and information recall (Figure 4). The process should be seen as a cycle, where each outcome can in turn influence the use of online health information and shape interactions between patients and providers. We note that this model is merely a *starting point,* and future research should complement this with relevant theoretical concepts that were unconsidered in our study.

This study also adds to the existing literature on computer-tailored health communication. Tailored online health information tools, typically providing recipients with *content* adapted to their individual characteristics, needs, and/or preferences have shown to be more effective than nontailored information on a wide range of patient outcomes, however typically with small effects [67-71]. Such tailored communication interventions may have provided personally relevant *content* but have possibly overlooked individual preferences for *how* this information should be presented. Moreover, the way in which content is processed highly depends on how this information is delivered [72]. Even though effects were small, this study suggests that tailoring the mode of information presentation to individual preferences, abilities, and/or learning styles could enhance the effectiveness of online health information interventions. Future research combining different tailoring strategies, such as content tailoring and mode tailoring, is warranted to tell whether effect sizes of tailored health communication interventions can be improved.

### Strengths, Limitations, and Future Research Directions

This study is the first to translate mode tailoring research to a clinical population of patients with cancer. Major strengths are its longitudinal character (ie, following patients from before to during and after their hospital visit) and the combination of observational data (ie, website tracking data and video observations) and patient-reported outcomes. We were able to include over 240 patients in our trial, which is highly unique given the emotional burden that newly diagnosed patients with cancer experience and including patients at this stage is challenging. About half (53.3%, 276/517) of patients declined participation. Although there was no age and gender difference between participating and nonparticipating patients, it is possible that those with varying education or health literacy levels were more or less likely to participate. Other RCTs employing educational interventions in cancer care report inclusion rates approximately between 25% and 60% [73-75]. Given that our inclusion rate was 47%, we believe that our carefully crafted inclusion protocol made it possible to reach the desired sample size in a difficult-to-reach population (see the *Methods* section).

Previous tailoring studies examining modes of information presentation have been useful in identifying which message features yield effects on outcomes and in unraveling the theoretical mechanisms explaining these effects [17,18,76]. However, these findings must be translated to different clinical contexts to establish whether such interventions have added value in different real-life settings. Several null findings in this study, concerning website experience outcomes (eg, communication self-efficacy) and consultation-related outcomes (eg, question asking), cannot be compared with the previous research. Below we discuss possible reasons why mode tailoring did not produce similar effects in this study’s clinical setting compared with previous experimental studies [17,18].

The first explanation is the age discrepancy between our previous experimental work and this RCT. *Younger* patients, that is, those aged younger than 65 years, were for the greater part not represented in the experimental studies as they included younger adults between the ages of 25 and 45 years [17,18]. Moreover, as participants in the experimental studies were recruited via an online research panel, these older participants were likely to be healthier and have more internet experience than clinical older patients in this RCT.

The second explanation is the high topic involvement of patients and therefore high personal relevance of the website content, irrespective of how it was presented. The ELM suggests that information is more likely to be processed deeply when a person’s interest for certain information is high, resulting in greater effects on outcomes (eg, information recall) [20]. Our sample consisted of newly diagnosed patients who were directed to a website about the specific clinic they were referred to, with information about their specific condition. Additionally, information avoiders might be underrepresented in our sample, as they might be less inclined to participate in our study. This might explain why the website was well used across all conditions (with only 9 patients not viewing the website) and why the perceived relevance of information did not differ between conditions. Consequently, it is possible that information was processed equally well from all website versions, revealing no differences in outcomes between conditions. Relatedly, it could be that some patients in the nontailored conditions received information in a way that coincidentally matched their preferences, attenuating effects of the mode-tailored website on outcomes. A previous tailoring study showed that when standardized information (by chance) corresponded to individual information needs, this was just as effective as tailored information [77]. Alternative explanations could be that the life-threatening nature of the disease (cancer), the emotionally charged moment (diagnosis), or a combination of these two elevated the perceived relevance and, consequently, website use. In this study, patients received the information while awaiting a final diagnosis and treatment plan, which is a phase in which information needs are the highest [26]. Future research could investigate whether mode tailoring has added value for clinical patients with a less life-threatening disease (eg, asthma, diabetes, and hypertension).

The third explanation is the uncontrolled setting of a field trial. In experimental studies, participants are exposed to stimulus materials, such as websites, and asked about these materials immediately afterward. In our field study, patients received a link to the website and the baseline questionnaire several days before their hospital visit. This allowed for patients to fill out the baseline questionnaire assessing website experience variables (eg, website satisfaction, anxiety, and communications self-efficacy) on different days before their visit. Moreover, patients varied in how often and how long they consulted the website. The variability concerning *when* their answers were recorded and *how* they used the website could have diluted the observable effects (eg, knowledge scores), if present. Although it would have been ideal to standardize study procedures even more, this is difficult, and perhaps unfruitful, in a clinical field study with patients with cancer.

In a similar vein, our sample consisted of a heterogeneous group of patients dealing with different cancer types (ie, colorectal, esophageal, or stomach cancer) with varying health trajectories before their appointment at the outpatient clinic. Although we imposed a strict randomization procedure that showed that patients did not differ on patient background characteristics, the sample heterogeneity could have attenuated the observable effects. We managed to recruit a relatively large sample for a difficult-to-reach clinical population, but it remains possible that we were unable to detect some of the hypothesized effects owing to a lack of statistical power. As a solution, qualitative approaches (eg, observations of or interviews with patients who have used the website intervention) might give a more meaningful and in-depth analysis of how the website was used, by whom, and whether this had added value for patients. This might be especially true for health communication interventions where small effect sizes are expected, and it remains difficult to obtain large, relatively homogeneous samples.

This study design did not include a no-information control group. A no-information control group would be useful to examine whether offering online preparatory information in general would have added value, but we considered that it would add little insight into mode tailoring effects. Furthermore, including a no-information control group would significantly reduce the number of patients in each condition given that data collection was limited to a maximum period of 3 years, further limiting statistical power to detect group differences. Although a no-information control group was not included, the results show that all the variables that predicted knowledge and recall were associated with the website (website involvement, website comprehension, and time spent on the website) and, therefore, imply that additional information sources surrounding the consultation can benefit patients.

In conclusion, we believe that there are many fruitful directions for future research. In addition to the suggestions we have already made, future research could for instance explore the relative importance of online information provision compared with offline, more traditional methods of information provision in different contexts and patient populations. To optimize information provision to all patients, researchers should continue to explore the added benefit of providing online preparatory information to patients (eg, in the form of hospital websites and patient portals), how specific features of the internet (eg, modality and interactivity) can be used to tailor information to patients, and whether different tailoring strategies (eg, content, mode, and cultural tailoring) are effective for patients regarding different types of patient outcomes (eg, evaluative, cognitive, psychosocial and behavioral) and in different patient populations (eg, high emotionally charged settings and non–life-threatening chronic diseases).

### Conclusions

This RCT showed that higher use of online health information to prepare for consultations benefits patients’ knowledge levels before a hospital visit. Higher knowledge, in turn, facilitates information processing and results in better information recall from medical consultations and knowledge acquisition from online information after their visit. Moreover, viewing online health information in a tailored presentation mode (ie, textual, visual, and/or audiovisual) increased younger patients’ satisfaction with the health website and reduced their anxiety after consultation, but not for older patients. The results are important in refining existing theoretical frameworks of patient-provider communication in an eHealth era.

## Abbreviations

ELM: elaboration likelihood model
GIOCA: Gastro-Intestinal Oncological Centre Amsterdam
LC4MP: limited capacity model of motivated mediated message processing
NPIRQ: Netherlands Patient Information Recall Questionnaire
RCT: randomized controlled trial
STAI-6: 6-item version of the State-Trait Anxiety Inventory
